# Comparison of the 7th and 8th Edition of the UICC/AJCC TNM Staging System in Primary Resected Squamous Cell Carcinomas of the Lung—A Single Center Analysis of 354 Cases

**DOI:** 10.3389/fmed.2019.00196

**Published:** 2019-09-04

**Authors:** Christina Neppl, Manuel D. Keller, Amina Scherz, Patrick Dorn, Ralph A. Schmid, Inti Zlobec, Sabina Berezowska

**Affiliations:** ^1^Institute of Pathology, University of Bern, Bern, Switzerland; ^2^Department of Medical Oncology, Inselspital, Bern University Hospital, University of Bern, Bern, Switzerland; ^3^Department of General Thoracic Surgery, Inselspital, Bern University Hospital, University of Bern, Bern, Switzerland

**Keywords:** TNM, staging, lung cancer, squamous cell carcinoma, NSCLC

## Abstract

**Background:** The AJCC/UICC TNM (tumor, node, metastasis) classification is a standardized system for the description of anatomical extent and stage grouping of solid malignant tumors and is regularly updated. We aimed at testing the new 2017 8th edition of the TNM classification (TNM8) compared to the former 2009 7th edition (TNM7), in pulmonary squamous cell carcinomas (pSQCC).

**Methods:** We analyzed a clinico-pathologically well-annotated Western single-center cohort of 354 consecutive pSQCC, resected 2000–2013, without previous neoadjuvant therapy. Patients with a clinical history of SQCC of other organs were excluded to reliably exclude lung metastases. Patients in whom TNM was unclear due to multiple tumor nodules were excluded. We reevaluated all pathological records and slides and retrospectively validated pleural invasion for all cases. Raw data of our cohort are provided as Supplementary Material.

**Results:** The stage distribution according to TNM7 was as follows: IA (2009): 59 (16.7%), IB: 75 (21.2%), IIA: 71 (20.1%), IIB: 53 (15.0%), IIIA: 79 (22.3%), IIIB: 7 (2.0%), IV: 10 (2.8%). Staging the cases according to TNM8, 7/354 (2.0%) cases were down-staged, 154 (43.5%) were upstaged; most pronounced between stages IIA(TNM7) and IIB(TNM8), and IIB(TNM7) and IIIA(TNM8). Both staging systems showed significant prognostic impact for overall survival, disease free and disease specific survival and time to recurrence, without significant differences regarding goodness-of-fit criteria (Akaike Information Criterion and Schwarz Bayesian Criterion).

**Conclusion:** In conclusion, we show a significant stage migration between tumors staged using TNM7 and TNM8, without benefit regarding prognostication in our cohort of primary resected pSQCC.

## Introduction

The tumor-node-metastasis (TNM) classification aims at standardizing the description of the anatomical extent of solid malignant tumors, resulting in the classification of patients into standardized stage groups for determining their prognosis and the resulting treatment plan. Effort is made to continuously refine classification criteria. The current 8th edition of the TNM classification (TNM8) was published by the Union for International Cancer Control (UICC) in 2017 (1), as a revision of the 2009 7th edition (TNM7) (2) and concurrently published by the American Joint Committee on Cancer (AJCC), with effective implementation in 2018 (3). The adaptations in TNM8 are based on recommendations provided by the Staging and Prognostic Factors Committee (SPFC) of the International Association for the Study of Lung Cancer (IASLC) published in 2014–2016, informed on the analysis of large international case collections with data supplied by 46 institutions in 19 countries (4–7).

Major changes of TNM8 compared to TNM7 applicable to pulmonary squamous cell carcinomas (pSQCC) are (a) more refined tumor size cut points in every T-category, using 1 cm intervals up to the size of 5 cm, (b) the classification of main bronchus involvement as T2, with removal of the 2 cm distance from the carina as a limit to separate pT2 and pT3 tumors, (c) classification of partial as well as total atelectasis as T2, and (d) regarding diaphragm invasion as a T4 instead of T3 descriptor. Mediastinal pleural invasion was removed from the criteria of T3 definition due to infrequent use. Furthermore, distant metastases outside the chest cavity are now subdivided according to number of metastatic foci.

TNM stage groupings are altered accordingly (7). There are no changes in the N-category.

Several groups from Asia and North America have validated the data in mixed cohorts of non-small cell lung cancer, showing mostly improved prognostication for TNM8 (Table 1) (8–14). Of note, in a large population based European study on resected T3N0 NSCLC improved prognostication of TNM8 depended on histological tumor typing (15). SQCC is usually underrepresented in mixed cohorts (Table 1).

**Table 1:** External validation studies of TNM8 vs. TNM7.

	**Cohort**	***N* total cohort**	**Improved discrimination of prognostic groups using TNM8 vs. TNM7**	**N SQCC**	**Subanalysis on SQCC**	**Country**
Jung et al. (12)	pN0M0 primary resected NSCLC (1999–2012)	1,316	No (equal)	385 (29.3%)	n.a.	Korea—single institution
Yun et al. (14)	≤ IIIB Primary resected NSCLC (2006–2012)	3,950	yes	1,069 (27.1%)	n.a.	Korea—single institution
Chen et al. (9)	Primary resected NSCLC (2006–2015)	2,043	yes	378 (18.8%)	n.a.	China—single institution
Sui et al. (10)	Primary resected IA-IIIA (TNM7) NSCLC (2005–2012)	3,599	yes	1,094 (30.4%)	n.a.	China—two institutions
Yang et al. (13)	NSCLC (2004–2013)	368,367 (cT) 177,409 (pT)	yes	n.a.	n.a.	North American—NCDB
Chansky et al. (11)	NSCLC^*^ (2000–2012)	612,534 (cT) 182,616 (pT)	yes	25%	n.a.	North American—NCDB
Okami et al. (8)	Resected NSCLC (2010)	18,973	yes	3,776 (20.1%)	n.a.	Japan—JJCLCR
Blaauwgeers et al. (15)	pT3N0M0 (TNM7) NSCLC (2010–2013)	683	Depending on tumor type	257	For pT3 due to 2 nodules poorer prognosis for SQCC	Netherlands—IKNL and PALGA

* *If surgically treated, only primary resected tumors included*.

*NSCLC, non-small cell lung cancer; NCDB, National Cancer Data Base; IKNL, Netherlands Comprehensive Cancer Organization; PALGA, Nationwide Network and Registry of Histopathology and Cytopathology in the Netherlands; JJCLCR, Japanese Joint Committee of Lung Cancer Registry Database*.

In the present study we aimed to compare the new TNM8 with the previous TNM7 regarding stage migration and prognostication in a clinico-pathologically very well-annotated Swiss single-center pure and large cohort of pSQCC.

## Patients and Methods

### Patient Cohort

The patient cohort consisted of all consecutive patients with primary resected pSQCC, without neoadjuvant treatment, resected between January 2000 and December 2013, and diagnosed at the Institute of Pathology, University of Bern. The cohort was assembled according to the pathology files, and subsequently validated by re-checking clinical hospital files and contacting general practitioners as described elsewhere (16).

In order to include only SQCC originating from the lung and reliably exclude metastases, patients with previously or concomitantly diagnosed SQCC of other organ systems were excluded. We excluded patients who died within 30 days following surgery (perioperative death) from survival analyses in order to avoid bias by surgery related short-term mortality. This retrospective single center study was approved by the Cantonal Ethics Commission of the Canton of Bern (KEK 200/14), which waived the requirement for written informed consent. The study was conducted and is reported according to the REMARK-guidelines (17).

### Histological Tumor Typing Including Immunohistochemistry

Histological tumor type and histological staging-parameters were reevaluated for each tumor by two pathologists specializing in lung pathology (CN, SB) according to the current WHO classification guidelines, using routine histomorphology and immunohistochemistry (IHC) (18).

IHC was performed using an automated immunostainer (Bond III, Leica Biosystems, Muttenz, Switzerland). Deparaffinized tissue-sections were rehydrated, followed by antigen retrieval. Endogenous peroxidase activity was blocked with H2O2 solution (Leica Biosystems). The anti-TTF1 clone 8G7G3/1 (Cell Marque, Rocklin, CA, USA) was used at a dilution of 1:400. After antigen retrieval performed with Tris-EDTA, pH 9 for 30 min at 95°C, TTF1 was incubated for 15 min at RT. The polyclonal anti-p40 (Biocare Medical, Biosystems Switzerland AG, Switzerland) was used at a dilution of 1:100. After antigen retrieval performed with Tris-EDTA, pH 9.0 for 30 min at 100°C, anti-p40 was incubated for 30 min at RT. Subsequently, samples were incubated with the secondary antibody using the Bond Polymer Refine Detection Kit with 3-3′-diaminobenzidine–DAB as chromogen (Leica Biosystems), counterstained with hematoxylin, and mounted in Aquatex (Merck, Darmstadt, Germany).

### Staging

For the purpose of this study all tumors were re-staged according to TNM7 and TNM8 (1, 2).

Information on tumor size and location was re-evaluated, extracted from the pathology reports and validated using clinical files and histological slides. Pleural invasion is stage relevant in tumors up to 3 cm, and was re-assessed for the purpose of this study in all cases according to current recommendations by reviewing all histological slides per tumor and if necessary additionally performing elastica-van-Gieson stains according to standard protocols, thereby visualizing tumor confined by or breaking through pleural elastic fibers (19). Lymph-node staging was validated using the IASLC lymph node map (20).

### Clinical Parameters

Clinical parameters were collected as previously reported (21). In short, data was collected from the patients' hospital files. We contacted patients' general practitioners for additional data provision, especially regarding progression, survival data and cause of death.

Time to recurrence (TTR) was defined as the time elapsed from the day of resection to loco-regional or metastatic recurrence or disease-specific death. Disease-specific survival (DSS) was measured from the day of resection to disease-specific death. Patients who died due to unrelated causes or secondary malignancies were censored at the time of death. Disease-free survival (DFS) was defined as the time elapsed from the day of resection to loco-regional or metastatic recurrence or death of any cause. Overall survival (OS) was assessed from the day of resection to death of any cause.

### Statistical Analysis

IBM SPPS Statistics 24 (IBM Corporation, Armonk, USA) was used for analysis. Group comparisons were performed using crosstabs, Chi^2^-tests and Fisher's exact tests, where appropriate. Kaplan-Meier curves and log-rank tests were calculated for univariate survival analysis. Cox regression analysis was used for multivariate analysis. The significance level for all statistical tests was set at 0.05.

The Akaike Information Criterion (AIC) and Schwarz Bayesian Information Criterion (SBC) were used for comparison of the goodness-of-fit between TNM7 and TNM8. Both methods adjust the −2 log likelihood statistics for the number of parameters in the model and number of observations used. Lower values of AIC and SBC indicate superior model fit with the “best” model showing the lowest values for both.

## Results

### Patient Cohort

The initial cohort included 385 patients. Twenty-three cases without clear morphological diagnostic criteria for SQCC (keratinization or intercellular bridges) were excluded: 7 due to TTF1 positivity, suggestive of adenocarcinoma, 12 due to no or only weak p40 expression in a screening approach as well as in whole slide re-evaluation, insufficient for final diagnosis of SQCC (18), and four cases due to an alternative histological diagnosis on re-evaluation. Insufficient clinical data and inconsistency between clinical and pathological staging lead to the exclusion of eight cases. Finally, 354 cases were available for analysis. All data on the patient cohort is provided in Table S1.

There were 302 (85.3%) males and 52 (14.7%) females, with a median age of 69 years at the time of resection (range 43–85).

Survival data were available for finally 256 patients, after thirteen patients were excluded due to perioperative death. Survival data up to 5-years after resection was included for analysis, due to the multimorbidity of the cohort. Median DFS was 44 months (95% CI = 36–41 months) and median OS was 47 months (95% CI = 38–43 months). Median TTR (data available for 213 patients) was 47 months (95% CI = 37–43 months).

Of note, 34/88 (38.6%) deaths were unrelated to pSQCC (e.g., heart attack, secondary lung adenocarcinoma).

### Tumor Characteristics

Median tumor size was 4.5 cm (range 0.8–15 cm). Pleural invasion was seen in 94/354 (26.6%) patients, with penetration through the pleural elastic membrane (PL1) in 43 (12.1%), tumor cells on the pleural surface (PL2) in 28 (7.9%), and invasion of the parietal pleura (PL3) in 23 (6.5%) patients.

Ipsilateral mediastinal lymph node metastases (pN2) were detected in 40 (11.3%) patients, hilar-parenchymal lymph node metastases (pN1) in 110 (31.1%), and 204/354 (57.6%) patients had no nodal disease (pN0). There was no patient with contralateral or station 1 lymph node metastases (pN3), therefore no patient classified as UICC stage IIIC. Ten (2.8%) patients had distant metastases.

### Shifts of the T-Descriptor Between the TNM7 and TNM8

Detailed data on the frequencies of the T-descriptors according to TNM7 and TNM8 are provided in Table 2A.

**Table 2:** Comparison of the T-descriptor and Stage distribution as assessed by TNM7 vs. TNM8 (*N* = 354).

**A**		**pT (TNM7)**							**Total (%)**
		**pT1a**	**pT1b**	**pT2a**	**pT2b**	**pT3**	**pT4**		
pT (TNM8)	pT1a	6	0	0	0		0	0	6 (1.7)
	pT1b	26	0	0	0		0	0	26 (7.3)
	pT1c	0	48	0	0		0	0	48 (13.6)
	pT2a	0	0	65	0		4	0	69 (19.5)
	pT2b	0	0	48	0		4	0	52 (14.7)
	pT3	0	0	0		54	27	0	81 (22.9)	
	pT4	0	0	0	0		45	27	72 (20.3)
Total (%)		32 (9.0)	48 (13.6)	113 (31.9)	54 (15.3)	80 (22.6)	27 (7.6)	354 (100)	
**B**		**Stage (TNM7)**							**Total (%)**
		**IA**	**IB**	**IIA**	**IIB**	**IIIA**	**IIIB**	**IV**	
Stage (TNM8)	I A 1	4	0	0	0	0	0	0	4 (1.1)
	I A 2	21	0	0	0	0	0	0	21 (5.9)
	I A 3	34	0	0	0	0	0	0	34 (9.6)
	I B	0	47	0	2	0	0	0	49 (13.8)
	II A	0	28	0	1	0	0	0	29 (8.2)
	II B	0	0	71	15	4	0	0	90 (25.4)
	III A	0	0	0	35	55	0	0	90 (25.4)
	III B	0	0	0	0	20	7	0	27 (7.6)
	IV A	0	0	0	0	0	0	7	7 (2.0)
	IV B	0	0	0	0	0	0	3	3 (0.8)
Total (%)		59 (16.7)	75 (21.2)	71 (20.1)	53 (15.0)	79 (22.3)	7 (2.0)	10 (2.8)	354 (100)

*Gray squares depict cases with stage migration*.

In the new TNM8 classification, the pT1-category was further subdivided by adding the pT1c-subdivision that is equivalent regarding size descriptors to the TNM7 pT1b subdivision (>2–3 cm). Accordingly, all 48 TNM7 pT1b cases were included in TNM8 pT1c.

The TNM7 pT1a was split between the TNM8 subdivisions pT1a (≤1 cm) and pT1b (>1–2 cm). Accordingly, 6/32 (18.8%) of patients from the TNM7 pT1a group remained in the TNM8 pT1a group, 26/32 (81.2%) were staged as TNM8 pT1b.

The size categories were also shifted in the TNM7 pT2a group (>3–5 cm), now encompassing TNM8 pT2a (>3–4 cm) and pT2b (>4–5 cm). This resulted in 48/113 (42.5%) TNM7 pT2a tumors to be up-staged to pT2b. Because the size equivalence of the TNM7 pT2b subdivision with the TNM8 pT3 category (>5–7 cm), all TNM7 pT2b tumors were up-staged to pT3.

The only tumors down-staged were from the TNM7 pT3 category (Table 2A). Down-staging of 7/80 (8.8%) TNM7 pT3 cases resulted from the removal of the 2 cm distance from the carina as a limit to separate pT2 and pT3 tumors with main bronchial involvement. Four tumors were down-staged to TNM8 pT2a (Pat ID 248, 249, 250, 251), another 3 to pT2b (Pat ID 252, 255, 254; see Table S1). One patient (Pat ID 253, see Table S1) was down-staged to pT2b due to reclassification of tumors with total atelectasis from TNM7 pT3 to pT2. Forty-five/80 (56.3%) TNM7 pT3 category tumors were up-staged to pT4, now encompassing all tumors over 7 cm diameter.

In summary, pT was significantly up-staged in 147/354 (41.5%) patients and down-staged in 8/354 (2.3%) patient using TNM8.

### Shifts of Tumor-Stage Between TNM7 and TNM8

Detailed data on the frequencies of UICC-stages according to TNM7 and TNM8 are provided in Table 2B.

The TNM7 stage IA was subdivided into TNM8 stages IA1 (pT1a), IA2 (pT1b), and IA3 (pT1c). Thus, the stage-distribution of the 59 N0-M0 tumors among 80 pT1-tumors mirrored the pT1-subdivisions. Of 75 TNM7 IB tumors, 28 (37.3%) were up-staged to TNM8 IIA, all of them due to up-staging from TNM7 pT2a to TNM8 pT2b. N1 lymph-node involvement was staged as IIA in pT1-pT2a tumors and as IIB in pT2b tumors according to TNM7. In TNM8, any tumors up to pT2b with N1-lymph-node involvement are staged as IIB. All TNM7 IIA tumors were thus up-staged to TNM8 IIB. In total, 7/354 (2%) tumors were down-staged using TNM8 due to down-staging TNM7 pT3 cases through removal of atelectasis and the 2 cm distance from the carina as a limit to separate pT2 and pT3 tumors, in node-negative (TNM7 IIB to IB: Pat. ID 248, 250; to IIA: 254) and N1-node-positive tumors (TNM7 IIIA to IIB: Pat. ID 249, 251, 252, 253; see Table S1).

Thirty-five/53 (66%) TNM7 IIB tumors were up-staged to IIIA, 15 N0-tumors due to up-staging TNM7 pT3 to pT4, 20 N1-tumors due to up-staging TNM7 pT2b to pT3. pT3-N2 tumors are newly staged as IIIB instead of IIIA. This resulted in up-staging of 16 tumors, among them 9 were up-staged from pT2b to TNM8 pT3. Four tumors were size dependently up-staged from TNM7 pT3 to pT4. In total 20/79 (25.3%) tumors were up-staged from TNM7 IIIA to TNM8 IIIB. There was no change in distributions of stages IIIB and IV.

In summary, 154/354 (43.5%) tumors were up-staged and 7/354 (2.0%) down-staged using TNM8.

### Survival Analysis and Prognostic Value of TNM7 and TNM8

**Figure 1:**
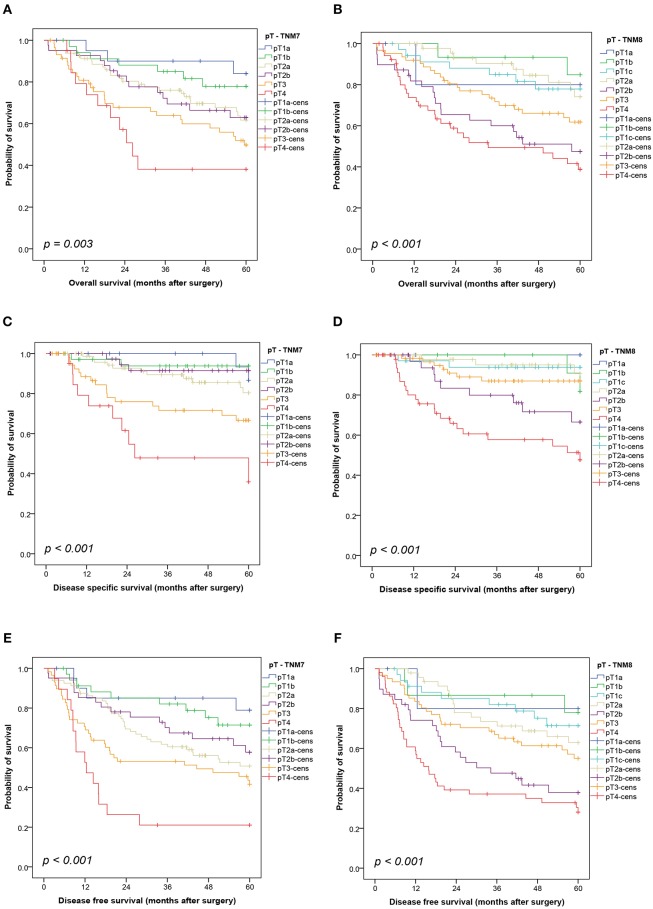
Kaplan-Meier curves showing the overall survival **(A,B)**, disease-specific survival **(C,D)** and disease-free survival **(E,F)** by pT-descriptor according to the seventh and eighth editions of the TNM classification. Comparisons were conducted using a log-rank test. cens, censored.

**Figure 2:**
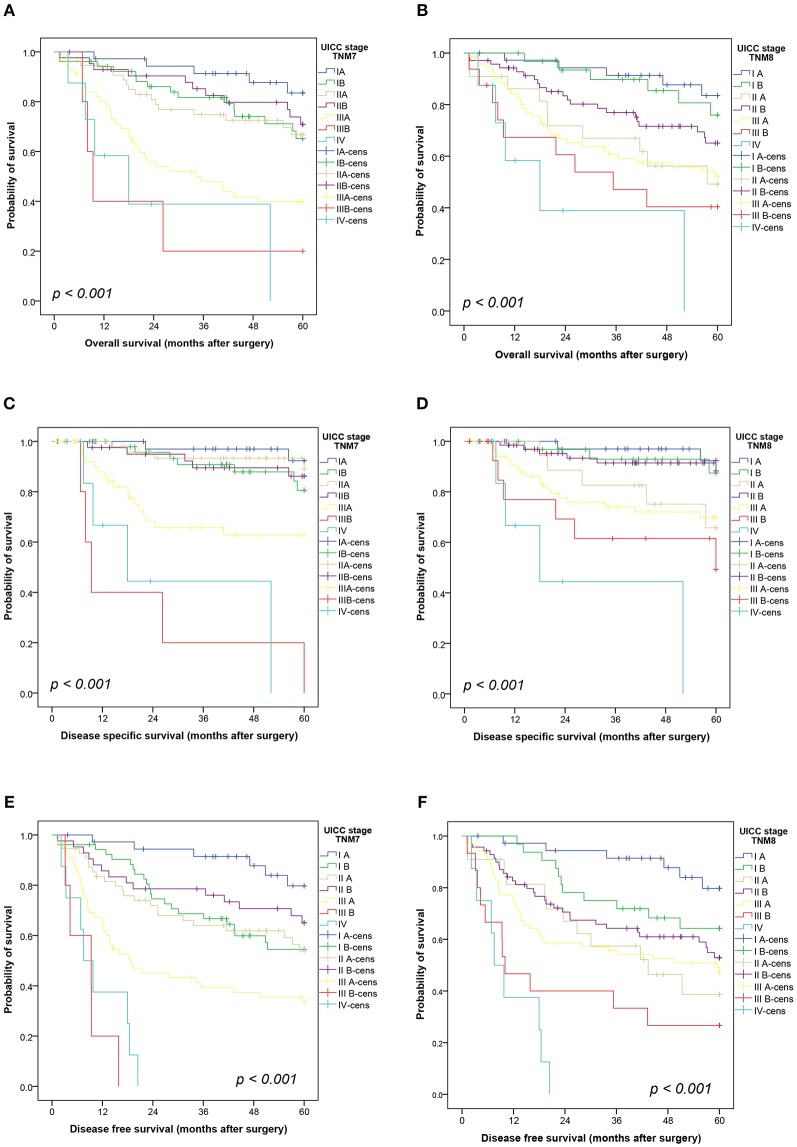
Kaplan-Meier curves showing the overall survival **(A,B)**, disease-specific survival **(C,D)** and disease-free survival **(E,F)** by stage according to the seventh and eighth editions of the TNM classification. Comparisons were conducted using a log-rank test. cens, censored.

Survival data were available for 256 patients. Both staging systems showed significant prognostic impact regardless if assessment was conducted using the isolated pT-descriptor (Figure 1) or the combined UICC stage (Figure 2) for OS (pT-TNM7 *p* = 0.003, pT-TNM8 *p* < 0.001; UICC both *p* < 0.001), DSS (both TNMs pT and UICC stage *p* < 0.001), DFS (both TNMs pT and UICC stage *p* < 0.001), and TTR (both TNMs pT and UICC stage *p* < 0.001). The Kaplan-Meier curves for TTR are provided as Figure S1.

Although TNM staging showed prognostic significance in overall analysis, patients with TNM8 UICC stage IIA seemed to have a worse outcome compared to stage IIB, though not statistically significant, for OS (*p* = 0.204), DSS (*p* = 0.066), and DFS (*p* = 0.350) (Figure 2). This was mirrored in seemingly swapped curves in the Kaplan-Meier plots for pT2b- and pT3-grouped tumors in TNM8 only (Figure 1). The reason for the discrepancy between TNM7 and TNM8 was the shift of 62% (32/52) TNM7 pT3 tumors into the TNM8 pT4 category due to tumor size >7 cm. The TNM7 pT2a group was subdivided between TNM8 pT2a, showing a prognosis similar to the pT1 categories, and the TNM8 pT2b group with very poor survival. The poor survival of the 36 patients shifting from TNM7 pT2a to TNM8 pT2b is not easily explained. In particular, there are no differences regarding age, or gender distribution between patients with TNM8 pT2b and pT3 tumors. Incomplete resections were more prevalent in the pT3 group (11/51 vs. 1/38, *p* = 0.026), as was administration of adjuvant chemotherapy (18/42 vs. 4/33, *p* = 0.044).

We used the Akaike Information Criterion (AIC) and the Schwarz Bayesian Criterion (SBC) as parameters for goodness-of-fit. The only minimal variance of AIC and SBC denotes no significant difference in model fit between TNM7 and TNM8 in our cohort (Table 3).

**Table 3:** Comparison of goodness-of-fit criteria between TNM7 and TNM8.

		**TNM7**	**TNM8**
OS	AIC	890.698	900.33
	SBC	905.562	915.194
DFS	AIC	1142.98	1159.392
	SBC	1159.397	1175.809
DSS	AIC	421.923	436.518
	SBC	432.628	447.223

*TNM7, TNM 7th ed.; TNM8, TNM 8th ed.; AIC, Akaike Information Criterion; SBC, Schwarz Bayesian Information Criterion; OS, overall survival; DFS, disease free survival; DSS, disease specific survival*.

## Discussion

In the present study, we compared the TNM7 system (2) with the recently published updated TNM8 (1) in a clinico-pathologically well-annotated, large, Swiss single-center cohort of pSQCC. Although there was a stage migration regarding the pT-descriptor and UICC stage in a significant number of cases, both TNM7 and TNM8 were prognostically highly significant regarding OS, DSS, DFS, and TTR. Presumably due to the relatively small cohort size, we could not demonstrate a statistically significant superiority of TNM8 comparing the two systems.

The strength of our study is the histologically homogeneous cohort and the re-validated clinico-pathological parameters for each patient. Especially in studies including tumors resected over a long period of time, it must be considered that pathological workup, histological assessment and diagnostic criteria have changed during the last years. For the present study, we re-evaluated all cases according to current WHO 2015-diagnostic criteria for pSQCC (18).

Additionally, SQCC in the lung may also represent metastases from extrathoracic primaries. Because risk factors for the development of SQCC of the lung and oral cavity are virtually identical, some patients suspected to suffer from pSQCC may in fact harbor lung-metastases from oral SQCC. As there are no histological or immunohistochemical differences between SQCC from different organ systems, care should be administered in assembling cohorts. In a unique approach, we have assured true primary pulmonary disease in our cohort by excluding all patients with synchronous SQCC elsewhere or a history thereof, thereby reliably excluding possible metastatic disease.

Even though the database used for developing the recommendations for TNM8 contained a very large number of 94,708 lung cancer patients, only 30,018 patients had sufficiently assessable information to settle a pathological pT descriptor, and data was gained from 35 sources in 16 countries. Half of the included patients originated from European sources, but no patients from Switzerland were included (5).

Several validation studies were subsequently published as summarized in Table 1. We consider our study valuable as an additional source for elucidating specific aspects of the TNM-classification in real life, by concentrating on one specific histological subtype with very consistent pathological and clinical data that were reassessed and validated for this study.

Our cohort size is comparable to the SQCC content of previous single-center studies validating TNM8 (9, 12). In a Chinese cohort of resected NSCLC including only 18.5% (378/2043) SQCC Chen and co-workers reported a marginally superior performance of TNM8 regarding prognostication for the whole cohort (9). A group from South Korea found no differences between TNM7 and TNM8 assessing the prognostic value of the pT-descriptor in primary resected node-negative lung carcinomas without specifying histological type (12). In a two-center study on a Chinese population, prognostication using TNM8 was superior to TNM7 in a cohort or primary resected NSCLC including 1094 SQCC (10). Studies investigating large national databases validate the TNM8 applicability in discriminating patient groups (8, 11, 13). To date, none of the studies validating TNM8 vs. TNM7 reported a subanalysis for pSQCC.

The evaluation of the National Cancer Data Base (NCDB; cT: 612,534; pT: 182,616) rendered no significant prognostic differences between clinically staged IIA (T2b) and IIB (T3) groups (11). Only 30% of the total cohort was pathologically staged (*N* = 182.616), and the overlap between T2b/T3 and IIA/IIB was lost in this subgroup. The authors speculate that imprecise radiological tumor measurement may account for the findings (11). There was also no significant difference in survival between cIIA and cIIB in a large Japanese Database study including 18,973 patients (8). An overlap between IIA and IIB or T2b and T3 groupings was also present in our cohort. Although both TNM7 and TNM8 were prognostically highly significant regarding OS, DSS, DFS, and TTR in overall analysis, patients with TNM8 UICC stage IIB had a worse outcome compared to stage IIA, mirrored by a worse outcome of patients with TNM8 pT2b vs. pT3 tumors in TNM8 only, although those differences were not statistically significant. Our data provide further validation for splitting TNM7 pT2a tumors into TNM8 pT2a and pT2b, as TNM8 pT2a patients showed a better survival, but we could not sufficiently explain the poor prognosis of our TNM8 pT2b group e.g., by differing clinico-pathological patient characteristics. The finding warrants careful monitoring of the respective subgroups in subsequent studies.

One limitation of our study is the relatively small cohort size compared to nationwide databases, which is inherent in the single center approach and the narrowly defined study population. To alleviate this concern, we provide full raw data of our cohort to be available for subsequent meta-analyses (Table S1).

The pivotal studies establishing adjuvant chemotherapy in NSCLC were based on the 5th and 6th edition of the TNM classification (TNM5 and TNM6). Even though we show a significant stage migration regarding pT and tumor stage, the decision criteria for adjuvant chemotherapy (AC) remain unaffected. The indication for AC is mainly based on the nodal stage, which has not been amended since TNM5. The value of AC in patients with lymph node involvement (N1 and N2 disease) corresponding to stages II and III was confirmed by meta-analysis after more than two decades, translating into a 4–5% absolute increase in 5-year OS (22). This benefit was achieved with cisplatin-based doublets, administering at least 300 mg/m^2^ in 3–4 cycles. Most data support the use of vinorelbine in combination with cisplatin in the adjuvant setting. The role of AC in node-negative disease is less evident. A small OS benefit was observed in a subgroup analysis for node-negative patients with tumor diameters ≥ 4 cm (23, 24). Importantly though, tumor stage often presents one inclusion criteria for clinical studies, and adaptation of stage-defining parameters must be taken into account.

In conclusion, we show a significant stage migration between tumors staged using TNM7 and TNM8, without benefit regarding prognostication in our cohort of pSQCC.

## Data Availability

The datasets analyzed for this study can be found in the Supplementary Material of this manuscript.

## Author Contributions

CN and SB conceived and designed the study and wrote the manuscript. CN, MK, AS, PD, RS, IZ, and SB researched and analyzed data, and edited and reviewed the manuscript. SB takes full responsibility for the work as a whole, including the study design, access to data and the decision to submit and publish the manuscript. All authors gave final approval for publication.

### Conflict of Interest Statement

The authors declare that the research was conducted in the absence of any commercial or financial relationships that could be construed as a potential conflict of interest.

## Supplementary Material

Figure S1 Kaplan-Meier curves showing the time to recurrence by T-descriptor **(A,B)** and stage **(C,D)** according to the seventh and eighth editions of the TNM classification. Comparisons were conducted using a log-rank test. cens, censored.

Table S1 Complete data on the patient cohort (*N* = 354).
