# Supplementary figures and images for: Comparison of the 7th and 8th Edition of the UICC/AJCC TNM Staging System in Primary Resected Squamous Cell Carcinomas of the Lung—A Single Center Analysis of 354 Cases
Source: Front Med (Lausanne). 2019 Sep 4;6:196. doi: 10.3389/fmed.2019.00196 (PMC6737333; doi:10.3389/fmed.2019.00196)

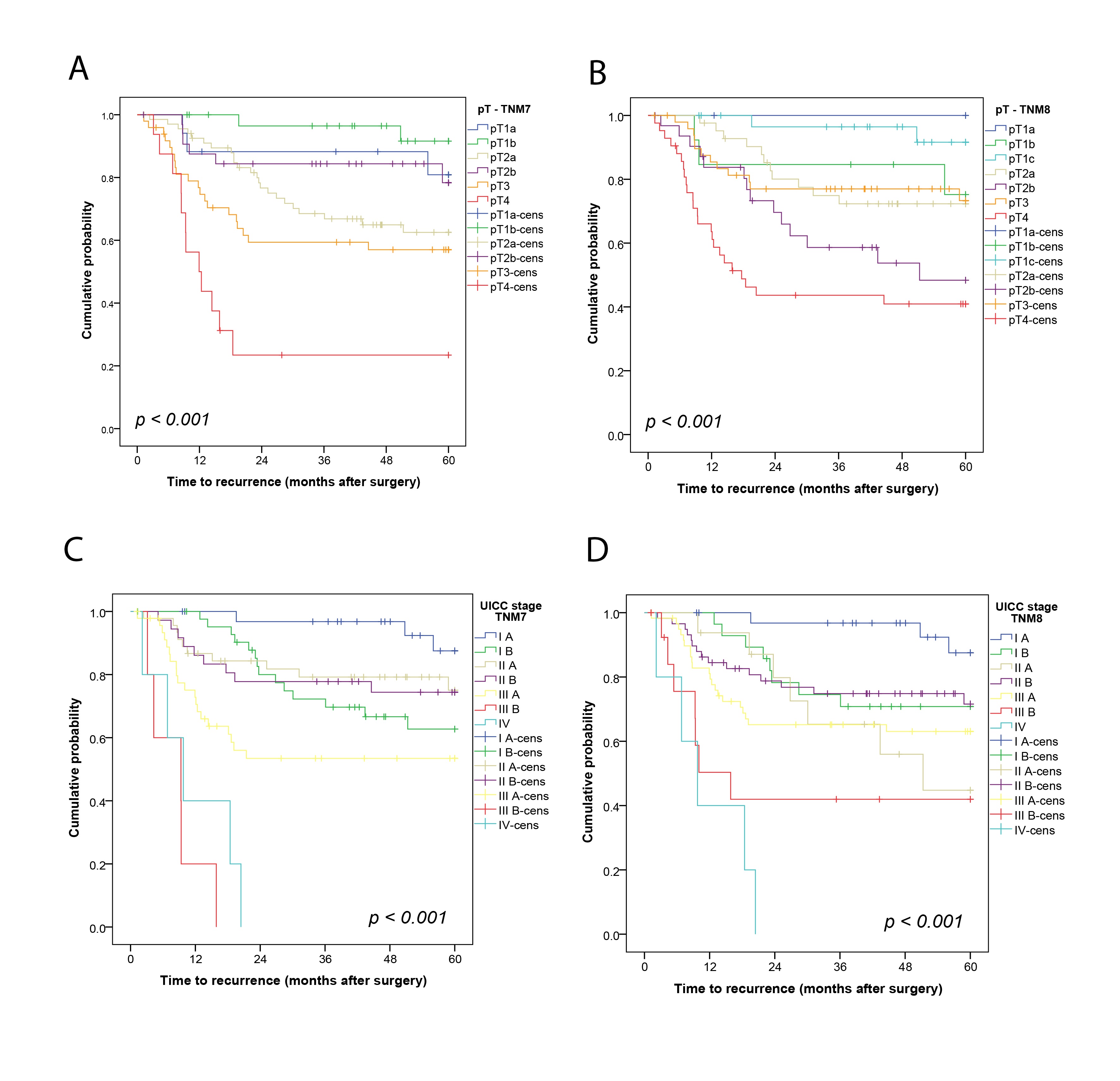

Supplement: Figure S1 — Kaplan-Meier curves showing the time to recurrence by T-descriptor (A,B) and stage (C,D) according to the seventh and eighth editions of the TNM classification. Comparisons were conducted using a log-rank test. cens, censored. [file Image_1.jpg]
